# Acupuncture for patients with glucagon-like peptide-1 receptor agonists-induced nausea and vomiting: A systematic review protocol

**DOI:** 10.1097/MD.0000000000020343

**Published:** 2020-05-22

**Authors:** Ning Ding, Linzhi Li, Xinyun Zhu, Xiaoying Huang, Lizhen Wang, Rensong Yue

**Affiliations:** a Hospital of Chengdu University of Traditional Chinese Medicine; b Chengdu University of Traditional Chinese Medicine, Chengdu, China.

**Keywords:** acupuncture, GLP-1 RAs, nausea, protocol, systematic review, vomiting

## Abstract

**Introduction:**

The glucagon-like peptide-1 receptor agonists (GLP-1 RAs) class agent has grown rapidly in the last decade due to its effects on lowering HbA1c and weight and the low possibility of hypoglycemia. However, GLP-1 RAs are not devoid of adverse effects among which nausea and vomiting rank first, which reduce adherence to treatment. Accumulated evidences proved that acupuncture can properly treat nausea and vomiting caused by various reasons. The study aims at assessing the safety and effectiveness exhibited by acupuncture treatment for patients with nausea and vomiting induced by GLP-1 RAs.

**Methods and analysis:**

Articles that have been identified via electronically searching databases of MEDLINE, Nature, PubMed, the Cochrane Library, WorldSciNet, EMbase, Science Online, AMED, China National Knowledge Infrastructure, the Wanfang Databse and China Biology Medicine Disc and the Chongqing VIP Chinese Science and Technology Periodical Database from their inception of to December 31, 2019 will be incorporated into the systematic review. The review only adopts Chinese and English. It will also pay attention to searching resources of qualified studies, relevant conference proceedings, potential reference list, as well as related system reviews. Two researchers will take charge of completing the selection of research, the extraction of data as well as the assessment of research quality independently. A random- or fixed-effects model will be employed to synthesize data combining the heterogeneity test. The primary outcomes will be nausea and vomiting, seen from the objective and self-reported assessment. Data analysis will be performed via the RevMan 5 software, and GRADE will help to assess the evidence level. The heterogeneity level will determine whether the random-effects model or the fixed-effects model will be used. The 2 categories will adopt risk ratio (RR) or odds ratio (OR) and 95% confidence interval (CI). Continuous variables will adopt the weighted mean difference or standardized mean difference and 95% CI. Meta-analysis will not be conducted if no assessment, like subgroup analysis, is able to explain existing meaningful heterogeneity. The subgroup analysis shall carefully consider each subgroup in certain case.

**Ethics and dissemination:**

The systematic review does not involve the evaluation of patients’ individual information or patients’ right; thus, there is no need to gain the approval from ethical institution. The article will be published in journals reviewed by peers and present at related conference.

Registration: Open Science Framework (OSF) Preregistration. 2020, April 8. osf.io/3fgu8

## 1 Introduction

Glucagon-like peptide-1 receptor agonists (GLP-1 RAs) act as the intestinal peptide mimetic of glucagon-like peptide-1 (GLP-1), one kind of incretin hormone derived from gut that inhibits glucagon secretion, stimulates insulin secretion, suppresses gastric emptying, as well as lowering food intake by reducing appetite.^[1]^ GLP-1 RAs have numerous advantages as an adjunctive therapy to antidiabetic drugs and lifestyle modification and in general, are well tolerated.^[2]^ However, gastrointestinal adverse events, nausea and vomiting as the most common ones which lead to high treatment discontinuation rate, are known to be associated with all GLP-1 RAs.^[3–6]^According to clinical researches, around 6% to 10% of GLP-1 RAs treatment discontinuation cases can be attributable to nausea, which accounts for 15% of the cases in which these drugs were tolerated only at reduced doses.^[7–10]^ Delayed gastric emptying could be one of the main mechanisms for the GLP-1 RAs-induced nausea and vomiting. Clinically, nausea and vomiting are more prevalent in patients with delayed gastric emptying compared to accelerated or normal.^[11]^ As an attractive option of diabetes and obesity, GLP-1 RAs deserve better application. If nausea and vomiting can be well treated via this class of agents, it will be used more properly and extensively.

Alternative treatment is adopted due to the side effects and low efficacy exhibited by antiemetics.^[12]^ Traditional Chinese medicine (TCM), characterized by holistic conception, syndrome differentiation and diseases occurrence and development prevention, refers to a systematic theory combining diagnosis, pathophysiology, and treatment based on the theory and practice accumulated for over 2000 years, of which acupuncture is a major aspect. Some studies have shown that acupuncture has the function of treating nausea and vomiting properly caused by many reasons. As concluded by the 1997 National Institute of Health Consensus Conference on Acupuncture, “promising results have emerged showing the efficacy of acupuncture in adult postoperative and chemotherapy induced nausea and vomiting.”^[13]^ Al-Sadi et al^[14]^ performed a random double-blind controlled study on 81 patients, finding that following the laparoscopic gynecological surgery, the acupuncture group saw the incidence rate of nausea and vomiting decreasing from 65% to 35%, relative to the placebo group. In China, acupuncture can be used to safely treat the nausea and vomiting of patients, and there is basically no adverse effect. The review aims at assessing the safety and effectiveness exhibited by acupuncture treatment for patients suffering nausea and induced by GLP-1 RAs.

## 2 Methods

### 2.1 Study registration

The protocol has been registered.

Registration: OSF Preregistration. 2020, April 8. osf.io/3fgu8. The protocol was written following the statement guidelines of Preferred Reporting Items for Systematic Reviews and Meta-Analyses Protocols (PRISMAP).^[15]^ Changes on the full review will be reported as required.

### 2.2 Inclusion and exclusion criteria for study selection

#### 2.2.1 Inclusion criteria

Inclusion criteria are all randomized controlled trials (RCTs) about the acupuncture therapy specific to nausea and vomiting induced by GLP-1 RAs. The review only adopts the language of English and Chinese.

#### 2.2.2 Exclusion criteria

Exclusion criteria are non-RCTs, quasi-RCTs, case series, reviews, and animal studies, explained as follows:

1. Non-RCTs
2. Quasi-RCTs
3. Evaluation studies on transcutaneous electrical nerve stimulation, laser acupuncture, dry needling, cupping or moxibustion
4. RCTs comparing various types of acupuncture
5. Case series
6. Reviews
7. Animal studies

### 2.3 Types of participants

Patients receiving short- or long-acting GLP-1 RAs treatment will be studied. No sex, ethnicity, or education restriction is there.

### 2.4 Types of interventions

#### 2.4.1 Experimental interventions

Experimental group will include patients receiving manual acupuncture, body acupuncture, electroacupuncture, and other types of acupuncture treatment, as well as those receiving treatment combined with acupuncture. The treatment frequency and duration are not restricted.

#### 2.4.2 Control interventions

Control group will include patients receiving control interventions like sham acupuncture, placebo acupuncture, herbs, western medicine, routine care, conventional therapy, or no treatment (waiting list control).

### 2.5 Types of outcome measures

#### 2.5.1 Primary outcomes

The primary outcomes will be nausea and vomiting, seen from objective and self-report assessment.

#### 2.5.2 Secondary outcomes

None.

## 3 Search methods for study identification

### 3.1 Electronic searches

Electronic searching will focus on databases of PubMed, the Cochrane Library, MEDLINE, EMbase, AMED, Nature, Science Online, WorldSciNet, the Wanfang Databse and China Biology Medicine Disc, and China National Knowledge Infrastructure, the Chongqing VIP Chinese Science, and Technology Periodical Database, with the temporal from the inception of database to December 31, 2019.

### 3.2 Other search resources

**Figure 1:**
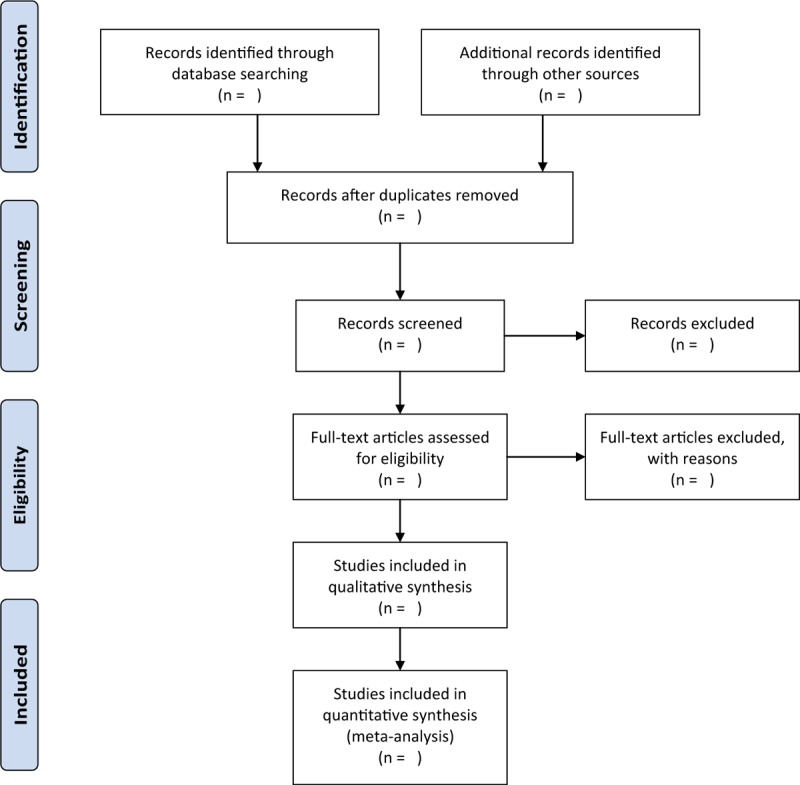
The research flowchart.

By manually retrieving and reviewing a reference list of those potential and qualified studies together with relevant system reviews, other RCTs’ location will be determined. Researchers will contact the trial author to obtain the latest clinical data for the convenience of ongoing RCTs. Besides, studies associated with the review will be identified via evaluating related conference proceedings. The research flowchart is shown in Figure 1.

### 3.3 Search strategy

Search terms will include 2 parts: acupuncture (like “acupuncture,” “acupuncture points,” “body acupuncture,” “manual acupuncture,” “electroacupuncture,” and “warm needle therapy”) and GLP-1 RAs-induced nausea and vomiting (like “glucagon-like peptide-1 receptor agonists,” “GLP-1 RAs,” “nausea,” “vomiting,” “nausea and vomiting,” “gastric emptying,” and “Drug-Related Side Effects and Adverse Reactions”). The study will use MeSH and text words. Appendix (Table 1) displays detailed search strategy for the PubMed database.

**Table 1:**
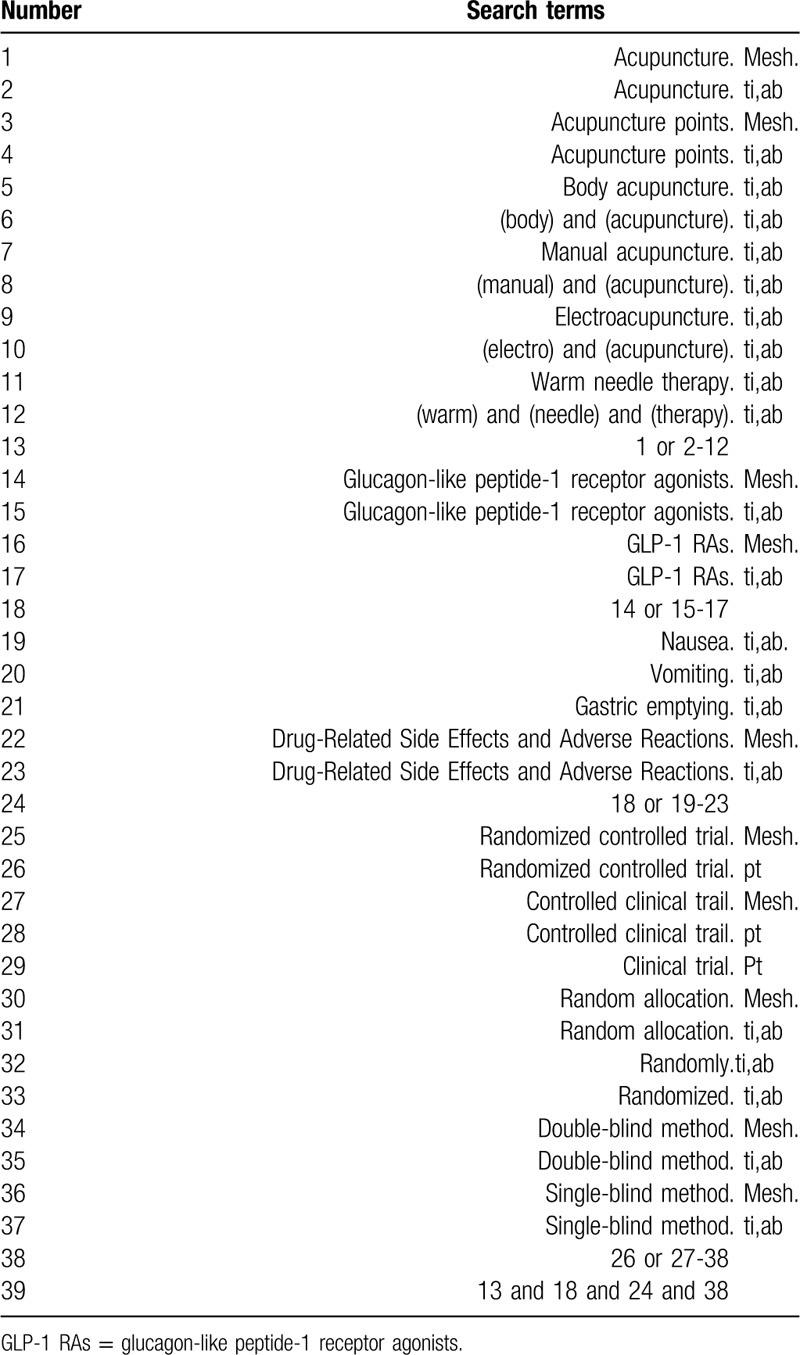
Search strategy for the PubMed database.

Terms of “zhenjiu,” “zhenci,” “dianzhen,” “wenzhen,” “exin,” “outu,” “GLP-1shoutijidongji,” “yigaoxuetangsuyangtai1shoutijidongji,” and “suijiduizhao” will be adopted from the Chinese database search. Proper modification will be performed on the strategy specific to other databases.

## 4 Data collection and analysis

### 4.1 Study selection

After the independent information extraction from literature included in the study, 2 researchers will input the extracted information into a unified statistical table of data. Ineligible studies as well as duplicate records will be first eliminated, followed by a review on the full text of those eligible ones for confirming their compliance to the abovementioned inclusion criteria. If the 2 researchers cannot come to an agreement, the final judgment will be made by the third researcher.

### 4.2 Data extraction and management

The following information will be extracted from each study: the reference ID, the first author, year of publication, GLP-1 RAs type, age of patient, intervention type, control intervention type, intervention group's sample size, intervention time, randomization, measure of outcome, allocation concealment method, blinding method, primary outcomes, follow-up duration, fund source and type, as well as a list of the Standards for Reporting Interventions in Controlled Trials of Acupuncture (STRICTA). The researchers will contact the author of study in the case of insufficient reported data. If negotiation cannot help to come to an agreement of the extraction of data, the final judgment will come to a third researcher.

### 4.3 Risk of bias assessment in included studies

Two researchers will independently adopt the Cochrane collaboration risk-of-bias assessment for assessing the quality of literature included in the review, together with completing the STRICTA checklist.^[16]^ Assessments include selective reporting, random sequence generation, incomplete outcome data, allocation concealment, blinding, as well as other possible biases. Related standards proposed in the Cochrane Intervention System Assessment Manual will be considered to classify risk of bias into three levels, low risk, high risk, and unclear risk. Discussion will be performed to resolve the discrepancy and a third research will take charge of making the final judgment if the 2 researchers cannot come into an agreement via discussion.

### 4.4 Treatment effect measures

Odds ratios (ORs) and MS and standardized mean difference (SMD) will assist in measuring the treatment effects specific to dichotomous outcomes and continuous outcomes, respectively. All these outcomes report 95% CIs.

### 4.5 Unit of analysis issues

The review will use the data of patients in RCTs. Each treatment will accept individual multiple meta-analyses with >1 acupuncture group within an RCT. Cross-over studies will use data obtained from the first sequence. The intervention and control groups will be analyzed by summarizing all controls’ results if there are many nonacupuncture controls.

### 4.6 Missing data management

The reason for the loss of data missed in the period of data screening and extraction will be identified here. Corresponding author will be contacted for obtaining missing data. With missing data unable to be obtained, available data will be analyzed only and the reason and effect of such exclusion will be explained.

### 4.7 Heterogeneity assessment

Meta-analysis will be carried out with the help of a random- or fixed-effects model. *Cochrane Handbook for Systematic Reviews of Interventions* describes that visually inspecting forest plot, a heterogeneity *x*^*2*^ test, as well as the Higgins *I*^*2*^ statistic can all help to assess the heterogeneity.^[17,18]^ The data will be pooled by a fixed-effects model with *P* value >.10 and the *I*^*2*^ value <50%, and by a random-effects model in other cases. When a set of studies exhibit an obvious heterogeneity, factors leading to the heterogeneity will be discussed, like the characteristics of patients and the variation degree in interventions. The heterogeneity will be evaluated via the subgroup analysis or the sensitivity group if applicable.

### 4.8 Reporting bias assessment

The biases of reporting will be assessed by virtue of a funnel plot if the meta-analysis includes over ten trials. The asymmetry exhibited by the funnel plot will be evaluated via the Egger and Begg tests, and *P* value <.05 means the publication bias is significant.

### 4.9 Data synthesis

Data analysis will rely on the RevMan 5 software (V. 5.3; Copenhagen: The Nordic Cochrane Center, The Cochrane Collaboration, 2014). The heterogeneity degree will help to confirm whether a random-effects model or a fixed-effects model will be used. The 2 categorical variables will adopt the index of RR or OR and 95% CI. Continuous variables will adopt the index of weighted mean difference or SMD and 95% CI. Meta-analysis will not be conducted if no assessment, like subgroup analysis, is able to explain existing meaningful heterogeneity. The subgroup analysis shall carefully consider each subgroup in certain case.

### 4.10 Subgroup analysis

Subgroup analyses will consider the heterogeneity exhibited by the type of acupuncture (manual acupuncture, body acupuncture, or electroacupuncture), the type of control (placebo or sham acupuncture, no acupuncture, medical treatment or conventional therapy), the acupoint, and the clinical difference.

### 4.11 Sensitivity analysis

For testing if review conclusions are robust, primary outcomes will receive a sensitivity analysis based on criteria involving the size of sample, the quality of heterogeneity, and the statistic model (whether it is a random-effects model or a fixed-effects model).

### 4.12 Grading the evidence quality

The evidence quality for obtained results will be assessed via the GRADE method.^[19]^ The assessment includes risk of bias exhibited by studies, the heterogeneity, evidence directness, estimate precision of effect, and publication risk of bias. Evidence will be divided into 4 categories considering the level, namely high risk, moderate risk, low risk, and very low risk.

### 4.13 Ethics and dissemination

The system review results will be published in journals reviewed by peers, disseminated at related conference or publications reviewed by peers. Aggregated published data will be used for excluding data of individual patient; thus, there is no need for obtaining the ethical approval or patients’ informed consent.

## 5 Discussion

GLP-1 RAs are highly praised especially due to the low risk of hypoglycemia, whereas the adverse effects, nausea, and vomiting, clinically bother patients. Antiemetics with the best efficacy are serotonin antagonists, dexamethasone, and droperidol, alone or combined. Even so, droperidol has no longer been used as the Food and Drug Administration of United States has recently warned that it could lead to cardiac arrhythmia.^[20]^ Due to the side effects and limited efficacy with antiemetics, alternative treatment appears. Nonpharmacological techniques such as acupuncture and acupressure have been the most widely recommended.^[21]^ TCM is used to treat symptoms rather than only disease itself. Acupuncture offers a new option which makes adverse effects no longer such hard-to-tackle problems. Konrad et al^[20]^ show that stimulation at the P6 acupoint could prevent or attenuate nausea and vomiting to some extent. Despite the continuous report of multiple RCTs of acupuncture for treating GLP-1 RAs-caused nausea and vomiting, there is no systematic evaluation on the cumulative evidence that proves its efficacy. The study will systematically review the effectiveness and safety of acupuncture treatment for patients suffering nausea and vomiting induced by GLP-1 RAs for the first time. Thus, for both clinical practice and further study, it seems that the resulting evidence of the review can contribute to some useful information which could effectively help patients, health policy makers, practitioners, as well as acupuncturists.

## Author contributions

**Conceptualization:** Ning Ding, Linzhi Li, Xiaoying Huang.

**Data curation:** Ning Ding, Linzhi Li, Xiaoying Huang.

**Formal analysis:** Xinyun Zhu, Xiaoying Huang, Lizhen Wang.

**Funding acquisition:** Ning Ding.

**Methodology:** Xinyun Zhu, Lizhen Wang.

**Project administration:** Ning Ding, Linzhi Li, Xiaoying Huang.

**Supervision:** Ning Ding.

**Writing – original draft:** Ning Ding, Linzhi Li.
